# The Drug Resources of the Colonies

**Published:** 1916-07-22

**Authors:** 


### The Drug Resources of the Colonies.

In his presidential address at the fifty-third annual meeting of the British Pharmaceutical Conference, Mr. David Hooper dealt with a subject with which nobody is more familiar than himself—namely, the drug resources of our overseas Dominions, notably India. "Imagine," he said, "what our supply of drugs and chemicals would be without our colonies. It should be a source of satisfaction to a chemist and druggist to know that the contents of his drawers and bottles are largely from plants and minerals obtained from British soil." India is especially rich in official and non-official drugs, and we have enjoyed her benefits ever since the time when our ancestors sailed to the East to bring "spices, precious stones, and drugges for the Poticaries." The fragrant gum-resins of former times, myrrh and frankincense, are still sorted, stored, and sold from the godowns of Bombay. Senna leaves are gathered from the irrigated lands of Tinnevelly and exported from Tuticorin. The deciduous forests of the Eastern and Western coasts yield nux vomica, more than sufficient to meet all demands. But scores of important drugs could be named which hail from India, and the drugs of India have for many years been an attractive field for chemical research. After alluding to the drug resources of Australasia and South Africa, Mr. Hooper said a few words on the topical subject of the cultivation of medicinal plants. In this connection he said there should be a central depôt for receiving, grading, and despatching the parcels, and a bureau for taking orders, executing them, and arranging prices and dates of delivery. Information should be published in the form of a calendar, with an exchange column to be consulted by those who wish to exchange or purchase plants and seeds. Finally he pointed out the importance of remembering that the British herb market was limited, and that those who engage in the supply should form a judicious knowledge of its requirements, regulate the price accordingly, and maintain the right standard of purity.

